# Web-Based Treatment Program Using Intensive Therapeutic Contact for Patients With Eating Disorders: Before-After Study

**DOI:** 10.2196/jmir.2211

**Published:** 2013-02-04

**Authors:** Elke D ter Huurne, Marloes G Postel, Hein A de Haan, Constance H.C Drossaert, Cor A.J DeJong

**Affiliations:** ^1^Tactus Addiction TreatmentEnschedeNetherlands; ^2^Nijmegen Institute for Scientist Practitioners in AddictionNijmegenNetherlands; ^3^University of TwenteDepartment of Psychology Health & TechnologyEnschedeNetherlands; ^4^Radboud University NijmegenBehavioural Science InstituteNijmegenNetherlands

**Keywords:** Eating disorders, eHealth, Internet, Web-based treatment, Intensive therapeutic contact, Program evaluation, Treatment effectiveness.

## Abstract

**Background:**

Although eating disorders are common in the Netherlands, only a few patients are treated by mental health care professionals. To reach and treat more patients with eating disorders, Tactus Addiction Treatment developed a web-based treatment program with asynchronous and intensive personalized communication between the patient and the therapist.

**Objective:**

This pilot study evaluated the web-based treatment program using intensive therapeutic contact in a population of 165 patients with an eating disorder.

**Methods:**

In a pre-post design with 6-week and 6-month follow-ups, eating disorder psychopathology, body dissatisfaction, Body Mass Index, physical and mental health, and quality of life were measured. The participant’s satisfaction with the web-based treatment program was also studied. Attrition data were collected, and participants were classified as noncompleters if they did not complete all 10 assignments of the web-based treatment program. Differences in baseline characteristics between completers and noncompleters were studied, as well as reasons for noncompletion. Furthermore, differences in treatment effectiveness, treatment adherence, and baseline characteristics between participants of the three major eating disorder diagnostic groups EDNOS (n=115), BN purging (n=24), and BN nonpurging (n=24) were measured.

**Results:**

Of the 165 participants who started the web-based treatment program, 89 participants (54%) completed all of the program assignments (completers) and 76 participants (46%) ended the program prematurely (noncompleters). Severe body dissatisfaction and physical and mental health problems seemed to have a negative impact on the completion of the web-based treatment program. Among the participants who completed the treatment program, significant improvements were found in eating disorder psychopathology (*F*=54.6, df = 68, *P*<.001, *d*=1.14). Body dissatisfaction, quality of life, and physical and mental health also significantly improved, and almost all of these positive effects were sustained up to 6 months after the participants had completed the web-based treatment program. Body Mass Index improved only within the group of participants suffering from obesity. The improvement in eating disorder psychopathology occurred in all three eating disorder diagnostic groups, and the percentage of completers did not differ significantly between these groups. Participants’ satisfaction with the treatment program, as well as with their therapist, was high, and participants indicated that they would recommend the program to other patients with eating disorders.

**Conclusions:**

The results of this study suggest that the web-based treatment program has the potential to improve eating disorder psychopathology in patients with different types of eating disorders.

## Introduction

Approximately 1% of the young female population suffers from bulimia nervosa (BN) and 0.3% from anorexia nervosa (AN). The prevalence of binge eating disorder (BED) is at least 1% of the adult population [1]. Furthermore, a large segment of the population suffers from an eating disorder not otherwise specified (EDNOS) [2,3]. Yet despite the severe psychological, physical, and social consequences of eating disorders, only 6% of the patients with BN and 33% of the patients with AN are treated by mental health care professionals in the Netherlands [1]. Patients often do not ask for help themselves because of, for example, feelings of shame, a lack of awareness, ambivalence about the need for treatment, or a positive attitude towards their eating disorder behavior [4-7]. Most patients have suffered from severe eating disorders for many years before they eventually receive treatment. The Internet, which offers widespread access to information and has resulted in increasing usage among individuals, has already proven to be a suitable medium to offer effective interventions for patients with different kinds of psychological disorders, including alcohol abuse [8-11], depression [12-14], anxiety disorders [13,14], posttraumatic stress [15], and panic disorder [16,17]. The advantages of web-based interventions, such as anonymity and 24-hour access from any location, fit the needs of patients with eating disorders as well [5,18-21]. However, the number of studies on web-based interventions for adult patients with eating disorders is limited: existing studies focus primarily on prevention rather than treatment [22-30]. Moreover, most studies involve self-help or minimal contact interventions [31-34], whereas research has shown that intensive contact with a therapist is more effective [8,13,35-37]. There are also web-based interventions that focus exclusively on weight reduction in overweight and obese adults but not on other relevant eating disorder characteristics [38-40].

To our knowledge, only three web-based treatments using intensive therapeutic contact have been studied. One intervention included a 3-month email therapy, consisting of one or two emails sent per week by an online therapist [41]. The results of a Randomized Controlled Trial (RCT) showed that this email therapy significantly reduced the number of patients fulfilling DSM-IV eating disorder criteria, compared to a waiting list control group. However, almost identical results were found for patients who participated in a writing intervention with minimal therapeutic contact [41]. Another intervention consisted of eight weekly 90-minute group chat sessions led by a trained therapist [42]. This intervention proved to be effective in improving body image and eating attitudes and behaviors in patients with high body dissatisfaction, compared to a control group. However, the improvements in the chat intervention group were not as significant as the improvements in a face-to-face treatment group at the end of treatment. Due to continued improvements in the chat intervention group, there were almost no significant differences between the chat intervention and the face-to-face treatment group at 6-month follow-up [42]. The third web-based intervention using intensive therapeutic contact that was studied included a 20-week Internet-delivered Cognitive Behavioral Therapy (CBT) program, using 25 scheduled asynchronous therapist feedback moments, which proved to be effective in patients with bulimic symptoms [43]. Participants of the web-based intervention group reported clinically relevant reductions in bulimic symptoms, and those reductions were substantially greater at posttreatment compared to the reductions in the bibliotherapy group and the waiting list control group. One year after the treatment, the differences between the web-based intervention and the bibliotherapy were no longer significant due to improvements in the bibliotherapy group [43].

Despite the mainly positive results of these studies, only the asynchronous, therapist-guided treatment program is available in the Netherlands. The recently published effects of this intervention have been studied in patients with high body dissatisfaction and bulimic symptoms, but not in patients with the different DSM-IV eating disorder diagnoses (AN, BN, and EDNOS, including BED). Patients with AN are even excluded from all three web-based treatments; all that exists is an Internet-based relapse prevention program for AN patients who have already been discharged from in-patient therapy [44] and a successful online intervention for the carers of AN patients [45]. To offer all eating disorder patients in the Netherlands the possibility to participate in a low threshold online therapy, we developed a new web-based intervention.

The intervention consisted of a website, an online forum, and a web-based treatment program. The website [46] was freely accessible for everyone and included concise information about eating disorders and related topics alongside a detailed description of the treatment program (sign-up procedure, content, aims, and costs). All visitors were free to decide whether they wanted to sign up for the web-based treatment program. Visitors to the website also had access to the online forum, where they could exchange ideas and experiences with fellow sufferers. In addition, registered participants of the web-based treatment program could log in to their personal online dossier via the website. During the treatment program, patients and therapists communicated asynchronously, solely via the Internet. Patients retained the same therapist, who could be identified by a name and a photograph in the patient’s online dossier. No face-to-face or telephone contact took place during the web-based treatment program, unless patients explicitly requested this. The intensive and personalized interaction between patients and therapists was an essential element of the program and set it apart from other online self-help programs. The asynchronous communication resembled email contact but took place within the framework of a safe and secure web-based application. Asynchronous contact was chosen because the time delay between the responses gave patients more autonomy to decide when to participate in treatment. They also had the opportunity to think carefully about the response they wished to give to their therapist. The content of the treatment program was based on the Dutch Multidisciplinary Guideline for Eating Disorders [7] and the latest insights regarding the treatment of eating disorders [6]. The online format, the design, and the technical aspects of the program were comparable with the successful web-based intervention for problem drinkers [8,47].

The aim of this pilot study was to evaluate adherence to, and the effectiveness of, the web-based treatment program and also patients’ satisfaction with the program and their therapist, respectively. Differences in adherence, appreciation, and the effects of the web-based treatment between patients with a different eating disorder diagnosis were also investigated.

## Methods

### Participants and Procedure

**Figure 1:**
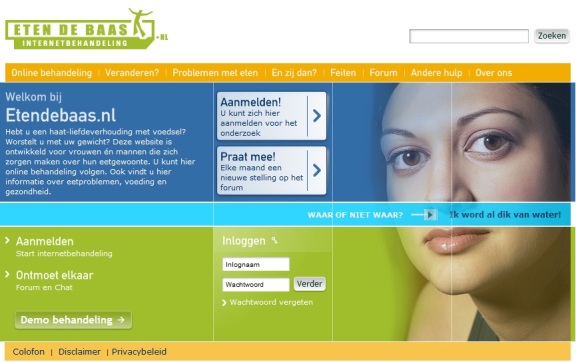
Homepage of the website.

The participants consisted of 165 adults who visited the website [46] (see Figure 1) and decided to sign up for the web-based treatment program between January 25 and December 31, 2010. The website was written for a general audience and all eating disorder diagnostic groups in order to reach a broad cross-section of the public and to recruit as many patients as possible for the web-based treatment program. The website and treatment program were promoted through various sources including relevant health care institutions, eating disorder-related websites, and a national press statement that resulted in newspaper, journal, and radio and television coverage. To sign up, participants provided personal data including their email address and information about their General Practitioner (GP) (to enable reimbursement of the treatment program by the health insurer). Participants were also informed about the terms and conditions of the treatment, after which they gave consent to participate by checking a box to indicate that they had read and understood the terms and conditions. Participants were then asked to choose a username and password and to fill in the baseline questionnaire. In total, 173 participants signed up for the treatment program although 8 (5%) did not start the program. Therefore, data from only 165 participants are included in the analysis.

### Intervention

**Figure 2:**
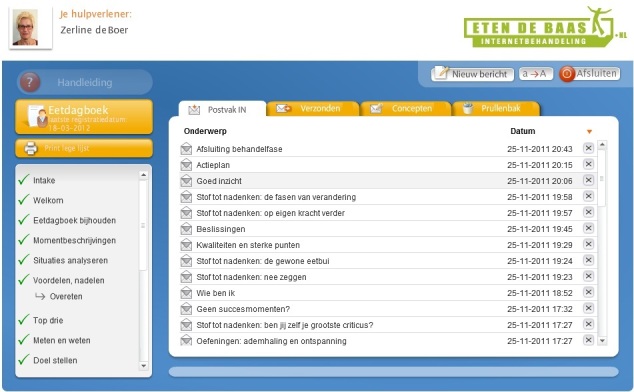
Participant's personal online dossier.

The structured, two-part, web-based treatment program was based on the principles of cognitive behavioral therapy (CBT) [19,48,49] and motivational interviewing (MI) [50,51]. Psycho-education, cognitive restructuring, self-control techniques, and exposure techniques were applied during the treatment program. The main aim of the program was to improve eating disorder psychopathology and to reduce body dissatisfaction. The average duration of the treatment program was about 15 weeks. Patients mostly maintained regular contact (1 or 2 times a week) with their therapist, and the therapist always responded within 3 working days to the messages of their patient. The therapists monitored the progress of the treatment program. In the event of irregular or limited contact (less than once a week), the therapist requested the patient to keep in touch regularly. Patients could access the web-based treatment program in their personal environment at any time they wished. By logging in to their personal dossier (see Figure 2), they had access to the messages sent by the therapist. These messages were personalized, although preprogrammed text parts were also used for the analogous parts of the program, eg, the explanation of an assignment. The therapist sent all assignments as attachments to their messages. Patients also had the option to request a face-to-face meeting or telephone contact.

The first part of the web-based treatment program included 4 assignments and at least 7 contact moments between the patient and the therapist, focusing on the analysis of the patient’s eating behavior. Patients were asked to register their daily eating behavior, analyze their eating situations, and describe the advantages and disadvantages of their eating problem. At the end of Part 1, the patients received personal advice from their therapist, who in turn obtained expert advice from the multidisciplinary team, which consisted of treatment staff, a doctor specialized in addiction, a psychiatrist, a psychologist, a dietitian, and supervisors. The second part started with setting a goal for eating behavior, exercising patterns, weighing, and compensatory behaviors. This part involved 6 assignments and at least 14 contact moments geared towards helping the patient reach the set goals and desired behavioral change. Examples of the assignments were: changing thought patterns, changing behavior patterns, improving the patient’s self-image, and writing a relapse prevention plan. If patients did not complete all 10 assignments, they were considered to be noncompleters.

All of the therapists involved had a Bachelors degree in nursing or social work or a Masters degree in psychology. All therapists followed an intensive training program that focused on motivational writing skills, the content and implementation of the treatment protocol, and the technical aspects of delivering the intervention. The training program included 2 days of theoretical information and practice-oriented assignments (eg, writing a response to a message received from a patient). After the training program, all therapists went on to complete a full treatment program with a test patient before they could start as an online therapist. They were subsequently supervised for a period of 3 months. If the trainers positively evaluated the therapists at this point, the therapists received a certificate for completing the training program. When the trainers judged a therapist to be unsuitable to work with the web-based treatment program, the training program was terminated prematurely.

### Outcome Measures

Participants completed online self-report measurements at baseline, posttreatment, 6-week and 6-month follow-ups. From the participants who prematurely ended the program, only baseline data were available as the measurement points linked to the treatment sessions.

The primary outcome measure of this pilot study was eating disorder psychopathology, which was assessed by using the Eating Disorder Examination Questionnaire (EDE-Q) [52,53]. The EDE-Q is a 36-item self-report scale that focuses on the previous 28 days to assess key behavioral and attitudinal features of eating disorders and the severity of the psychopathology of eating disorders. It consists of 4 subscales measuring 4 eating attitudes: Restraint, Eating Concerns, Shape Concerns, and Weight Concerns. The items were scored on a 7-point Likert-type scale ranging from 0 to 6. A higher score indicates a higher level of eating disorder psychopathology.

Secondary outcome measures were Body Mass Index (BMI), body dissatisfaction, physical health, mental health, and quality of life. BMI was measured by dividing the participants’ self-reported body weight in kilograms by the participants’ self-reported height in meters squared. Body dissatisfaction was measured using the 20-item Body Attitude Test (BAT) [54-57], which assesses the subjective perception and attitude of the participant towards his or her own body. The items were scored on a 6-point Likert-type scale ranging from 0 to 5. A higher score represents greater body dissatisfaction. A score above 36 indicates clinically significant disturbance. Physical health was assessed using the Maudsley Addiction Profile Health Symptom Scale (MAP-HSS): a 10-item self-report scale measuring physical complaints [58]. Each item was scored on a 5-point Likert-type scale ranging from 0 to 4. Because the MAP-HSS measures only general physical complaints, 15 additional eating disorder-specific physical complaints were added: dizziness/fainting; insomnia; hoarseness; sore throat; palpitations; diarrhea; constipation; hair loss/brittle hair; downy hair on face, arms, chest or back; fluid accumulation in the legs; dry/scaly skin; rapidly cold; dental problems; damaged back of the hand; and swollen glands. These items were scored on the same 5-point Likert-type scale. The total score of physical complaints was determined by dividing the sum of the scores on the 10 MAP-HSS items and the 15 additional items by the total number of items (n=25). A higher score represents a higher level of physical health problems. The 21-item Depression Anxiety Stress Scale (DASS-21) [59] measured the three related negative emotional states of depression, anxiety, and stress. Each item was scored on a 4-point Likert-type scale ranging from 0 to 3. A higher total score indicates a higher level of mental health problems. Quality of life was measured using the visual analogue scale of the EuroQol-5D (EQ-5D VAS) ranging from 0 (worst imaginable health state) to 100 (best imaginable health state). A higher score represents a higher quality of life.

Other measures at baseline included demographic characteristics, motivation for participating in the web-based treatment program, eating disorder diagnosis, previous treatment for eating disorders, and previous treatment for psychological problems. Demographic characteristics included age, gender, level of education, employment, and their daily routine. Participants were categorized as “higher educated” when they had a Bachelors or Masters degree. Participants’ motivation for participating in the web-based treatment program was measured with the question: “Why have you opted for the web-based treatment?” Possible answers were: (1) “I can do this on my own time”, (2) “I can do this from the confines of my own personal environment”, (3) “I can retain my anonymity”, (4) “I prefer contact via the Internet”, (5) “On the advice of a doctor/therapist”, and (6) “For another reason, namely …” (free text response). Eating disorder diagnosis was determined using self-report questions based on the DSM-IV-TR criteria of eating disorders. The MINI-Plus interview [60,61] was used as a guideline in the development of this self-report questionnaire.

At posttreatment, participants’ satisfaction with the program and their therapist was measured. Participants were asked which aspects of the treatment program they found most important, as well as how pleasant, personal, and safe they considered the communication with their therapist. Participants were also asked if web-based treatment was effective for them and if they would recommend the intervention to others. Participants had to rate the treatment program and their therapist on a scale from 0 (very low) to 10 (very high). Finally, they also had the possibility of providing additional comments.

### Analysis

A pre-post design was used to compare baseline data with outcome measures after completing the web-based treatment program. Multilevel modeling with SPSS, version 18, was used to determine improvement from baseline to posttreatment for the outcomes of interest. Repeated analyses of the outcome measures allowed for the inclusion of all participants, regardless of missing data, over time. For all outcomes, Cohen’s *d* effect sizes were calculated to analyze the strength of the observed effects [62]. Differences among the diagnostic groups and between completers and noncompleters were analyzed using chi-square tests for ordinal and nominal variables and one-way ANOVA (diagnostic groups) or independent sample *t* tests (completers/noncompleters) for scale variables.

## Results

### Participants

Table 1 presents the baseline characteristics of the 165 participants who enrolled in the pilot study. Of these participants, 98% (n=161) were female, 68% (n=113) were employed, and 42% (n=69) had a higher level of education. Of the participants (n=115), 70% fulfilled the criteria for EDNOS, 15% (n=24) for BN nonpurging, 15% (n=24) for BN purging, and 1% (n=2) for AN restrictive. Most participants had suffered from their eating disorder for many years, but 75 participants (45%) had never been in treatment before (eg, individual contact with a dietitian or a psychologist, admission to a clinic or hospital, or group therapy). However, 65% of the participants (n=108) had been in treatment for other mental health problems, mostly for depression or anxiety. The main reasons for participants to choose web-based treatment were that they could participate in the program on their own time and within their own personal environment.

We compared baseline characteristics among the three major diagnostic groups: EDNOS, BN nonpurging, and BN purging. Unfortunately, no separate analysis could be conducted for the AN diagnostic group because the pilot study included only 2 participants with AN. The differences between the three diagnostic groups, EDNOS, BN nonpurging, and BN purging, are presented in Table 1. Regarding demographic variables, we found that participants with BN purging were the youngest and participants with EDNOS the oldest. The percentage of participants who were employed was also the lowest in the BN purging group and the highest in the EDNOS group. For illness-related variables, significant differences were found for BMI, prior care for eating disorder, eating disorder psychopathology, quality of life, and mental and physical health. Participants in the BN purging group had received care for their eating disorder less frequently but experienced more physical and mental health problems than participants in the EDNOS and BN nonpurging groups. Eating disorder psychopathology was less severe among the participants with EDNOS; these participants had lower scores on the subscales “Restraint” and “Eating Concern”. Quality of life was highest among participants with BN nonpurging.

**Table 1:** Participant characteristics at baseline and differences between diagnostic groups.

Variable		Overall	EDNOS^a^	BN NP^b^	BN P^c^	Analysis	
		n=165	n=115	n=24	n=24	*F* value / χ^2^	*P*
**Female, n (%)**		161 (98%)	111 (97%)	24 (100%)	24 (100%)	1.71	.43
**Age (years), mean (SD)**		35.3 (11.0)	36.6 (10.2)	33.1 (11.4)	30.9 (13.1)	3.41	.04
**Employed, n (%)**		113 (68%)	87 (76%)	15 (63%)	11 (46%)	8.92	.01
**Higher education**		69 (42%)	53 (46%)	9 (38%)	6 (25%)	4.01	.14
**Regular daily routine, n (%)**		123 (75%)	87 (76%)	20 (83%)	15 (63%)	2.90	.23
**Prior care eating disorder, n (%)**		90 (55%)	68 (59%)	14 (58%)	6 (25%)	9.53	.009
**Prior care psychiatric problems, n (%)**		108 (65%)	77 (67%)	16 (67%)	13 (54%)	1.46	.48
**Body Mass Index, mean (SD)**		29.1 (9.2)	31.2 (9.4)	26.4 (6.7)	22.8 (5.3)	11.12	<.001
**Eating disorder psychopathology, mean (SD) ^d^**		3.4 (1.0)	3.2 (1.1)	3.8 (0.9)	3.8 (0.8)	5.42	.005
	Restraint	2.5 (1.6)	2.1 (1.5)	3.1 (1.6)	3.5 (1.3)	11.22	<.001
	Eating concern	3.0 (1.3)	2.8 (1.3)	3.3 (1.0)	3.4 (0.9)	4.14	.02
	Shape concern	4.3 (1.3)	4.2 (1.3)	4.5 (1.2)	4.3 (1.2)	0.67	.51
	Weight concern	4.0 (1.2)	3.9 (1.2)	4.3 (1.1)	4.0 (1.4)	1.28	.28
**Body dissatisfaction, mean (SD)** ^**e**^		60.5 (16.7)	61.0 (15.8)	62.1 (16.9)	55.5 (20.4)	1.21	.30
**Quality of life, mean (SD) ^f^**		59.4 (16.6)	59.1 (16.3)	67.4 (13.7)	53.3 (18.5)	4.61	.01
**Mental health, mean (SD) ^g^**		38.5 (19.8)	36.6 (20.0)	36.3 (16.0)	47.8 (19.7)	3.42	.04
**Physical health, mean (SD) ^h^**		2.1 (0.5)	2.0 (0.5)	2.1 (0.5)	2.4 (0.5)	6.33	.002

^a^EDNOS = eating disorder not otherwise specified.

^b^BN NP = bulimia nervosa nonpurging.

^c^BN P=bulimia nervosa purging.

^d^Eating Disorder Examination – Questionnaire (EDE-Q).

^e^Body Attitude Test (BAT).

^f^EuroQol-5D visual analogue scale (EQ-5D VAS).

^g^21-item Depression Anxiety Stress Scale (DASS-21).

^h^Total score consisting of Maudsley Addiction Profile Health Symptom Scale (MAP-HSS) and 15 additional eating disorder-specific physical complaints.

### Intervention Usage and Missing Data

**Figure 3:**
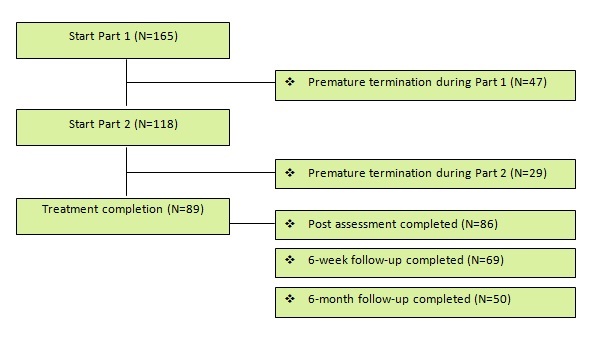
Participant flow.

More than half of the participants (n=89, 54%) completed all of the treatment sessions (completers), and 118 participants (72%) completed Part 1 of the program. Figure 3 provides an overview of the participant flow. Of the 76 participants (46%) who did not complete the entire program (noncompleters), the reasons for dropping out were mostly unknown. The 23 noncompleters (14%) who did provide a reason for stopping the treatment program mentioned a personal situation unrelated to the treatment (n=9), discomfort with the treatment protocol (n=6), discomfort with treatment via the Internet (n=4), satisfaction with the achieved results (n=2), or something else (n=2). The therapist discharged one participant due to the seriousness of her problem. The postassessment was completed by 86 of the 165 participants (52%), and the 6-week and 6-month follow-ups were completed by 69 participants (42%) and 50 participants (30%), respectively. There were no significant differences between the diagnostic groups in terms of the percentage of completers and noncompleters (χ^2^= 2.95; df = 2; *P*=.23).

### Completers Versus Noncompleters

We compared baseline characteristics between completers and noncompleters (Table 2). Almost all of the demographic characteristics did not differ significantly between those two groups, except for their daily routine. Completers more often had a regular daily routine than noncompleters. Regarding illness-related variables, noncompleters were more dissatisfied with their bodies and experienced a lower quality of life and more physical and mental health problems. The completers and noncompleters did not differ with regard to diagnosis, BMI, or eating disorder psychopathology.

**Table 2:** Differences in baseline characteristics between completers and noncompleters.

Variable		Completers	Noncompleters	Analysis	
		n=89	n=76	*t* value / χ^2^	*P*
**Female, n (%)**		88 (99%)	73 (96%)	1.38	.34
**Age (years), mean (SD)**		36.8 (11.2)	33.5 (10.5)	1.94	.054
**Employed, n (%)**		65 (73%)	48 (63%)	1.85	.17
**Higher education**		40 (45%)	29 (38%)	0.88	.35
**Regular daily routine, n (%)**		75 (84%)	48 (63%)	9.63	.002
**Prior care eating disorder, n (%)**		51 (57%)	39 (51%)	0.59	.44
**Prior care psychiatric problems, n (%)**		58 (65%)	50 (66%)	0.01	.93
**Body Mass Index, mean (SD)**		28.3 (7.9)	30.0 (10.5)	-1.19	.23
**Eating disorder psychopathology, mean (SD)** ^**a**^		3.4 (1.0)	3.5 (1.0)	-0.40	.69
	Restraint	2.6 (1.5)	2.3 (1.6)	1.32	.19
	Eating concern	2.9 (1.2)	3.1 (1.3)	-0.88	.38
	Shape concern	4.2 (1.3)	4.4 (1.2)	-0.79	.43
	Weight concern	3.9 (1.2)	4.1 (1.2)	-1.35	.18
**Body dissatisfaction, mean (SD)** ^**b**^		57.8 (15.6)	63.8 (17.5)	-2.33	.02
**Quality of life, mean (SD)** ^**c**^		62.2 (14.8)	56.3 (18.1)	2.28	.02
**Mental health, mean (SD)** ^**d**^		34.2 (17.9)	43.5 (20.8)	-3.10	.002
**Physical health, mean (SD)** ^**e**^		1.9 (0.5)	2.3 (0.5)	-4.54	<.001

^a^Eating Disorder Examination – Questionnaire (EDE-Q).

^b^Body Attitude Test (BAT).

^c^EuroQol-5D visual analogue scale (EQ-5D VAS).

^d^21-item Depression Anxiety Stress Scale (DASS-21).

^e^Total score consisting of Maudsley Addiction Profile Health Symptom Scale (MAP-HSS) and 15 additional eating disorder-specific physical complaints.

### Effectiveness of the Intervention

Table 3 presents the mean and standard deviations on the outcome measures of this pilot study. Eating disorder psychopathology significantly improved (*F*=54.6, df = 68, *P*<.001, *d*=1.14) with medium to large effect sizes (from *d*=.47 to *d*=1.17). We also found significant improvements in body dissatisfaction, quality of life, and mental and physical health. Almost all of the treatment effects were sustained at the 6-week and 6-month follow-up measurements; only the improvement in quality of life was no longer significant 6 months after completing the treatment program. For BMI, the improvements were significant only for participants with obesity (BMI > 30). However, the size of the effect was small (*d*=.20).

**Table 3:** Treatment outcomes for all participants.

Variable		Pretreatment		Posttreatment			Follow-up at 6 months			Overall effect^a^			
		Mean	SD	MD^b^	SD	*P*	MD^b^	SD	*P*	*F*	df	*P*	Effect size^c^
**Eating disorder psycho-pathology^d^**		3.4	1.0	1.4	1.2	<.001	1.2	1.4	<.001	54.6	68	<.001	1.14
	Restraint	2.5	1.6	0.9	1.8	<.001	0.7	1.5	.001	11.7	70	<.001	0.47
	Eating concern	3.0	1.3	1.6	1.4	<.001	1.2	1.5	<.001	55.6	70	<.001	0.95
	Shape concern	4.3	1.3	1.6	1.5	<.001	1.5	1.8	<.001	39.6	67	<.001	1.17
	Weight concern	4.0	1.2	1.4	1.5	<.001	1.3	1.6	<.001	41.6	68	<.001	1.11
**Body dissatisfaction** ^**e**^		60.5	16.7	15.3	13.6	<.001	14.5	17.1	<.001	40.8	67	<.001	0.86
**BMI** ^**f**^													
	< 18.5	16.9	1.3	-0.6	0.9	.29	-0.3	0.6	1.00	3.8	9	.05	0.02
	18.5-25	21.1	1.7	0.1	1.1	.99	0.7	1.8	.34	1.6	18	.22	0.39
	25-30	27.8	1.5	0.4	1.0	.66	0.7	1.9	.67	0.9	14	.48	0.46
	> 30	36.4	7.2	1.1	1.9	.01	1.5	2.1	.01	4.7	26	.01	0.20
**Quality of life** ^**g**^		59.4	16.6	-9.6	17.8	<.001	-6.8	20.5	.08	13.3	71	<.001	0.32
**Mental health** ^**h**^		38.5	19.8	12.8	16.1	<.001	11.0	17.9	<.001	21.8	70	<.001	0.56
**Physical health** ^**i**^		2.1	0.5	0.3	0.3	<.001	0.2	0.3	<.001	36.8	67	<.001	0.39

^a^Treatment outcomes were measured with Repeated Measures and Mixed Model analysis.

^b^MD = Mean Difference; positive MD scores indicate a decrease in baseline scores and negative MD scores indicate an increase in baseline scores.

^c^Effect sizes were measured with Cohen’s *d* using MD at 6-months follow-up and baseline SD.

^d^Eating Disorder Examination – Questionnaire (EDE-Q).

^e^Body Attitude Test (BAT).

^f^BMI indexes below 18.5 indicate underweight, 18.5 to 25 healthy weight, 25 to 30 overweight, and over 30 obesity.

^g^EuroQol-5D visual analogue scale (EQ-5D VAS).

^h^21-item Depression Anxiety Stress Scale (DASS-21).

^i^Total score consisting of Maudsley Addiction Profile Health Symptom Scale (MAP-HSS) and 15 additional eating disorder-specific physical complaints.

Analyses for individual diagnostic groups showed that eating disorder psychopathology significantly improved in the EDNOS group and that this improvement was sustained up to 6 months after completion of the web-based treatment program (Appendix 1). Participants from the EDNOS group also improved on all secondary outcome measures, and the effect sizes for all outcome measures were medium to large (from *d*=.45 to *d*=1.29). Participants from the BN nonpurging group also improved on most primary and secondary outcome measures with generally medium to large effect sizes, but the differences between the separate measuring moments (pretreatment and posttreatment, and pretreatment and follow-up at 6 months) were not significant for this group. Analyses for the participants from the BN purging group showed significant improvements in eating disorder psychopathology from pretreatment to posttreatment, but those improvements did not maintain at the 6-month follow-up. For all secondary outcome measures, positive trends were found at posttreatment; however, those improvements were not statistically significant and did not remain 6 months after treatment completion.

### Participant’s Satisfaction With the Intervention

Participants who completed the postassessment (n=86, 52%) were satisfied with the program and the contact they had with the therapist. Most participants (n=72, 84%) found web-based treatment to be an effective method for treating eating disorders and nearly all of the participants (n=78, 91%) stated that they would recommend the program to others. The support of the therapist was considered to be one of the most valuable and important components of the program. Most participants considered the online contact with the therapist to be pleasant (n=77, 90%), personal (n=61, 71%), and safe (n=82, 95%). The assignment “Changing thoughts” and the daily registration in the eating diary were also evaluated as very worthwhile and important. On a scale from 0 to 10, participants evaluated the treatment program with a 7.8 (SD 1.2) and their therapist with an 8.4 (SD 0.9). These evaluations did not differ among the three diagnostic groups (treatment program: *F*=0.01, df = 2, *P*=1.00; therapist: *F*=0.15, df = 2, *P*=.86). The most common criticisms were that the treatment program was too short (n=5), the therapist’s messages were sometimes too standard and impersonal (n=4), and the treatment sessions sometimes followed on from each other too quickly (n=3).

## Discussion

### Principal Results and Comparison With Prior Work

This pilot study showed that the web-based treatment program successfully changed the eating disorder psychopathology in patients with eating disorders and that these improvements were sustained at 6-week and 6-month follow-ups. Participants also indicated that they had become more satisfied with their bodies and that their physical and mental problems had decreased during the treatment program. Participants evaluated the program positively, with the support of the therapist rated as the most important element. Participants experienced the personal online contact with their therapist as pleasant, personal, and safe. On a scale from 0 to 10, they evaluated their therapist with an 8.4.

The improvement in eating disorder psychopathology in our pilot study is consistent with the results of other web-based interventions with intensive therapeutic contact, although our effect sizes seem to be somewhat larger [42-43]. We did not find a significant improvement in BMI for participants who were underweight (BMI < 18.5) and overweight (BMI = 25-30). Although the web-based treatment did not focus primarily on weight improvement, the underlying idea is that the improvement of eating disorder psychopathology will improve BMI. In the present study, there is only limited evidence for this among the participants with obesity (BMI > 30). Further research would be required to investigate how BMI changes can be achieved for all participants who are either underweight or overweight.

The attrition rate in our pilot study was 46%. Because of the linear design of our treatment program, nonusage attrition (program adherence) and dropout attrition (study adherence) were the same in our study. According to a systematic review on adherence to, and the effectiveness of, web-based therapies, it is often difficult to compare the attrition rate of interventions because of the large variation in the reporting of those results [63]. This also applies to the attrition rate of our study compared to the attrition rate of other web-based interventions for patients with eating disorders. For example, Paxton et al reported a nonusage rate of 16% for their web-based group chat intervention; however, they considered participants to be completers when they had attended only four of the eight intervention sessions [42]. In addition, Carrard et al reported a low dropout attrition rate (25%), but a high nonusage attrition rate (69%) [33]. Robinson and Serfaty, however, described only the dropout attrition rate in their study (47%). It is therefore not clear whether patients participated actively in the email therapy [41]. Ruwaard et al reported a nonusage attrition rate of 26% and a dropout attrition rate of 17% for their asynchronous web-based intervention [43]. One possible explanation for the lower attrition rates in this study might be selection bias due to the randomized design and the exclusion criteria of that study, with more than 60% of the participants stopping or being rejected even before randomization [43].

Completers and noncompleters differed significantly on several baseline characteristics. The baseline physical and mental health as well as participants’ satisfaction with their body seemed to play an important role in completing the web-based treatment program. Although little research has examined differences between completers and noncompleters of treatments for eating disorder patients (especially for web-based treatments), other studies have suggested that the risk of noncompletion increases with an increase in the severity of other health problems and comorbidity [64]. Therefore, the web-based treatment program can be seen as an important and accessible first step within the stepped-care principle, while participants who need more help will be referred to a more intensive form of treatment. However, further research into the factors that influence the completion of the treatment program is needed.

The web-based treatment was available for patients with all eating disorders; however, as expected based on prevalence rates, most of the participants (70%) fulfilled the criteria for EDNOS (including BED). Almost all of the other participants met the criteria of BN, with half of them belonging to the purging subtype and the other half to the nonpurging subtype. Only 2 participants fulfilled the criteria of AN. The low number of participants with AN can be a result of the recruitment strategy, as it focused on eating disorders in general and not on specific diagnostic groups. In addition, the lower prevalence of AN compared to the other eating disorder diagnostic groups might also be a reason for the limited number of patients with AN in our pilot study. However, the benefits of the web-based treatment program, such as the high degree of anonymity and the increased convenience, are particularly applicable for patients with AN. As such low-threshold forms of treatment for this particular target group are still missing from the current treatment services in the Netherlands, it is important to recruit more patients with AN for the web-based treatment program in the future. However, the recruitment should then be more focused on places where patients with this particular diagnosis can be found (eg, informative websites and forums for patients with AN, patient associations, health centers, general practitioners’ surgeries, and schools), and the message of the recruitment should also be more tailored for this target group.

As the pilot study included only 2 participants with AN, no separate analysis could be conducted for this group. Between the other diagnostic groups (EDNOS, BN purging, and BN nonpurging), we found significant differences regarding several demographic and illness-related variables. The differences in age and employment are not surprising as BN often occurs in young women (some of whom are still studying), while BED has a much broader age range. The differences in BMI and eating disorder psychopathology can be explained by the diagnosis, as participants with BN have compensatory behaviors that are related to body weight and eating disorder psychopathology. The study found no significant differences between the diagnostic groups related to treatment adherence and satisfaction with the program. In addition, eating disorder psychopathology improved within each diagnostic group. Therefore, the web-based treatment program seems feasible for patients with BN, as well as patients with EDNOS, including BED. However, some differences were evident among the diagnostic groups. These differences might be explained by the large differences in numbers between the three groups. It would be interesting to further investigate these differences among larger patient groups in order to draw reliable conclusions.

### Limitations

The pilot study has several limitations. As previously mentioned, almost half of the participants did not complete all of the treatment sessions provided through the program. Consequently, no posttreatment and follow-up data were available from the noncompleters, as these questionnaires were completed after the last treatment session. Therefore, we do not know whether these patients benefited from participating in the treatment program. We have chosen a linear model, as the treatment program is most effective with a specific order of treatment steps, and this model is also useful in working with homework assignments and tailored feedback. However, the lack of information about noncompleters of the intervention is a real disadvantage. We recently started a randomized controlled trial (RCT) to study the efficacy of the web-based treatment program. In the RCT, the web-based application has been modified so that the measurements are no longer linked to the treatment steps. Consequently, posttreatment and follow-up data will be available from both completers and noncompleters. The reasons for noncompletion and the characteristics of noncompleters, as well as their satisfaction with the treatment program and therapist, will also be investigated in the RCT.

Another limitation is that we cannot attribute the observed improvements exclusively to the web-based treatment program due to the nonrandomized design of the study. The RCT will therefore provide more insights into the effectiveness of this intervention. The results and our experiences of this pilot study were the foundation for the development of the RCT. As mentioned before, the web-based application has been modified to differentiate between the research questionnaires and the treatment steps. Study adherence and treatment adherence can therefore be distinguished. In addition, patients with BED will not be included in the EDNOS group in the RCT, but they will comprise an individual diagnostic group based on the BED DSM-IV-TR research criteria. Furthermore, patients with AN and male patients will be excluded, as these groups were a minority in this pilot study and it is not feasible to recruit enough patients within the RCT to be able to make statements about these individual patient groups. However, since the web-based treatment program has been developed for all patients with eating disorders, and we do not want to exclude male patients and AN patients, we will offer them the possibility to participate in the regular treatment program without participating in the RCT. Finally, in the RCT more attention will be paid to completing all research questionnaires to enlarge study adherence. The researcher will actively approach participants via email and phone and will request that they fill in all of the research questionnaires. This will also be stimulated with an incentive of €10.00 for each completed research questionnaire.

A last limitation of this pilot study concerns the reliability of some measurements. Although validated self-report instruments were used, clinical interviews might be more preferable. In addition, a direct measurement of the participants’ height and weight is more desirable than self-reported height and weight [65], but because all communication with participants occurred via the Internet, this was not possible in the present study.

### Conclusions

This pilot study indicated that the web-based treatment program with intensive therapeutic contact is an acceptable intervention for patients with eating disorders. Participants evaluated the program positively, and the results after completing treatment were promising. Eating disorder psychopathology and body satisfaction improved significantly, as did mental and physical health. The web-based treatment program also resulted in an improvement in the quality of life. A randomized controlled trial has recently been started to provide more scientific evidence for the efficacy of this web-based intervention.

## Multimedia Appendix 1

Treatment outcomes for individual diagnostic groups.

## Abbreviations

AN: anorexia nervosa
BAT: Body Attitude Test
BED: binge eating disorder
BMI: body mass index
BN NP: bulimia nervosa nonpurging
BN P: bulimia nervosa purging
CBT: cognitive behavioral therapy
DASS-21: Depression Anxiety Stress Scale
DSM-IV: Diagnostic and Statistical Manual of Mental Disorders, 4threvision
EDE-Q: Eating Disorder Examination Questionnaire
EDNOS: eating disorder not otherwise specified
EQ-5D VAS: EuroQol-5D visual analogue scale
MAP-HSS: Maudsley Addiction Profile-Health Symptom Scale
MI: motivational interviewing
MINI-Plus: Mini International Neuropsychiatric Interview Plus
RCT: randomized controlled trial
