# Supplementary material for: Web-Based Treatment Program Using Intensive Therapeutic Contact for Patients With Eating Disorders: Before-After Study
Source: J Med Internet Res. 2013 Feb 4;15(2):e12. doi: 10.2196/jmir.2211 (PMC3636210; doi:10.2196/jmir.2211)
Supplement: Supplementary file 1 [file jmir_v15i2e12_app1.pdf]

Treatment outcomes for individual diagnostic groups.

Variable	Pretreatment		Post-treatment			Follow-up at 6 months			Overall effect[a]			
	Mean	SD	MD [b]	SD	*P*	MD[b]	SD	*P*	*F*	df	*P*	Effect size[c]
**EDNOS group[d]**												
Eating disorder psychopathology[e]	3.2	1.1	1.4	1.3	<.001	1.3	1.3	<.001	40.5	48	<.001	1.26
Restraint	2.1	1.5	0.7	1.8	<.001	0.8	1.3	<.001	7.1	48	.001	0.53
Eating concern	2.8	1.3	1.6	1.5	<.01	1.3	1.5	<.001	44.5	51	<.001	1.00
Shape concern	4.2	1.3	1.7	1.6	<.001	1.7	1.7	<.001	31.5	49	<.001	1.29
Weight concern	3.9	1.2	1.5	1.5	<.001	1.5	1.6	<.001	32.4	48	<.001	1.28
Body dissatisfaction[f]	61.0	15.8	17.2	12.2	<.001	17.2	17.0	<.001	44.7	48	<.001	1.09
Quality of life[g]	59.1	16.3	-10.4	19.2	<.001	-7.3	22.3	.26	11.3	50	<.001	0.45
Mental health[h]	36.6	20.0	15.6	15.5	<.001	14.3	16.1	<.001	28.3	50	<.001	0.72
Physical health[i]	2.0	0.5	0.4	0.3	<.001	0.3	0.3	<.001	35.0	48	<.001	0.55
**BN NP group[j]**												
Eating disorder psychopathology[e]	3.8	0.9	1.0	1.2	.10	1.1	1.5	.41	4.8	7	.04	1.22
Restraint	3.1	1.6	1.1	1.5	.15	0.5	2.0	.98	3.3	10	.07	0.29
Eating concern	3.3	1.0	1.0	1.4	.19	0.9	0.9	.23	7.1	8	.01	0.92
Shape concern	4.5	1.2	0.9	1.4	.22	1.6	1.7	.13	3.7	8	.06	1.36
Weight concern	4.3	1.1	0.9	1.3	.20	1.2	1.9	.59	3.7	8	.06	1.02
Body dissatisfaction[f]	62.1	16.9	8.8	15.2	.41	13.3	16.3	.28	5.9	6	.03	0.79
Quality of life[g]	67.4	13.7	-3.7	10.0	.79	-7.8	13.2	.27	2.8	10	.10	0.57
Mental health[h]	36.3	16.0	3.8	19.8	.99	11.2	19.0	.40	4.9	10	.03	0.70
Physical health [i]	2.1	0.5	0.3	0.3	.04	0.2	0.3	.56	4.9	9	.03	0.38
**BN P group[k]**												
Eating disorder psychopathology[e]	3.8	0.8	1.8	1.1	<.001	0.1	1.3	1.00	27.7	11	<.001	0.09
Restraint	3.5	1.3	2.0	1.2	.001	1.0	1.6	.57	12.1	8	.002	0.80
Eating concern	3.4	0.9	1.7	1.1	.001	0.0	1.6	1.00	10.4	7	.005	0.00
Shape concern	4.3	1.2	1.8	1.4	.01	-0.3	1.8	1.00	17.6	9	<.001	0.25
Weight concern	4.0	1.4	1.5	1.1	.01	0.1	0.9	1.00	13.0	9	.002	0.00
Body dissatisfaction[f]	55.5	20.4	9.8	15.6	.22	-4.1	10.2	.90	3.1	8	.09	0.20
Quality of life[g]	53.3	18.5	-11.5	15.6	.16	-9.7	26.7	.92	7.8	10	.006	0.52
Mental health[h]	47.8	19.7	12.6	16.8	.18	-4.4	15.7	.98	3.1	10	.08	0.23
Physical health[i]	2.4	0.5	0.2	0.4	.36	0.0	0.5	1.00	2.0	7	.20	0.09

[a]Treatment outcomes were measured with Repeated Measures and Mixed Model analysis.
[b]MD = Mean Difference; positive MD scores indicate a decrease in baseline scores and negative MD scores indicate an increase in baseline scores.
[c]Effect sizes were measured with Cohen's *d* using MD at 6-months follow-up and baseline SD.
[d]EDNOS = eating disorder not otherwise specified; pre-treatment n = 115; post-treatment n = 64; follow-up at 6 months n = 34.
[e]Eating Disorder Examination – Questionnaire (EDE-Q).
[f]Body Attitude Test (BAT).
[g]EuroQol-5D visual analogue scale (EQ-5D VAS).
[h]21-item Depression Anxiety Stress Scale (DASS-21).
[i]Total score consisting of Maudsley Addiction Profile Health Symptom Scale (MAP-HSS) and 15 additional eating disorder-specific physical complaints.
[j]BN NP = bulimia nervosa non-purging; pre-treatment n = 24; post-treatment n = 11; follow-up at 6 months n = 9.
[k]BN P = bulimia nervosa purging; pre-treatment n = 24; post-treatment n = 10; follow-up at 6 months n = 6.
